# Human Intestinal Organoids: Promise and Challenge

**DOI:** 10.3389/fcell.2022.854740

**Published:** 2022-03-11

**Authors:** Jasin Taelman, Mònica Diaz, Jordi Guiu

**Affiliations:** ^1^ Cell Plasticity and Regeneration Group, Regenerative Medicine Program, Institut d’Investigació Biomèdica de Bellvitge–IDIBELL, L’Hospitalet de Llobregat, Spain; ^2^ Program for advancing the Clinical Translation of Regenerative Medicine of Catalonia, P-CMR[C], L’Hospitalet de Llobregat, Spain

**Keywords:** organoids, 3D models, intestine, human intestinal organoids, enteroids

## Abstract

The study of human intestinal biology in healthy and diseased conditions has always been challenging. Primary obstacles have included limited tissue accessibility, inadequate *in vitro* maintenance and ethical constrains. The development of three-dimensional organoid cultures has transformed this entirely. Intestinal organoids are self-organized three-dimensional structures that partially recapitulate the identity, cell heterogeneity and cell behaviour of the original tissue *in vitro*. This includes the capacity of stem cells to self-renew, as well as to differentiate towards major intestinal lineages. Therefore, over the past decade, the use of human organoid cultures has been instrumental to model human intestinal development, homeostasis, disease, and regeneration. Intestinal organoids can be derived from pluripotent stem cells (PSC) or from adult somatic intestinal stem cells (ISC). Both types of organoid sources harbour their respective strengths and weaknesses. In this mini review, we describe the applications of human intestinal organoids, discussing the differences, advantages, and disadvantages of PSC-derived and ISC-derived organoids.

## Introduction

**FIGURE 1:**
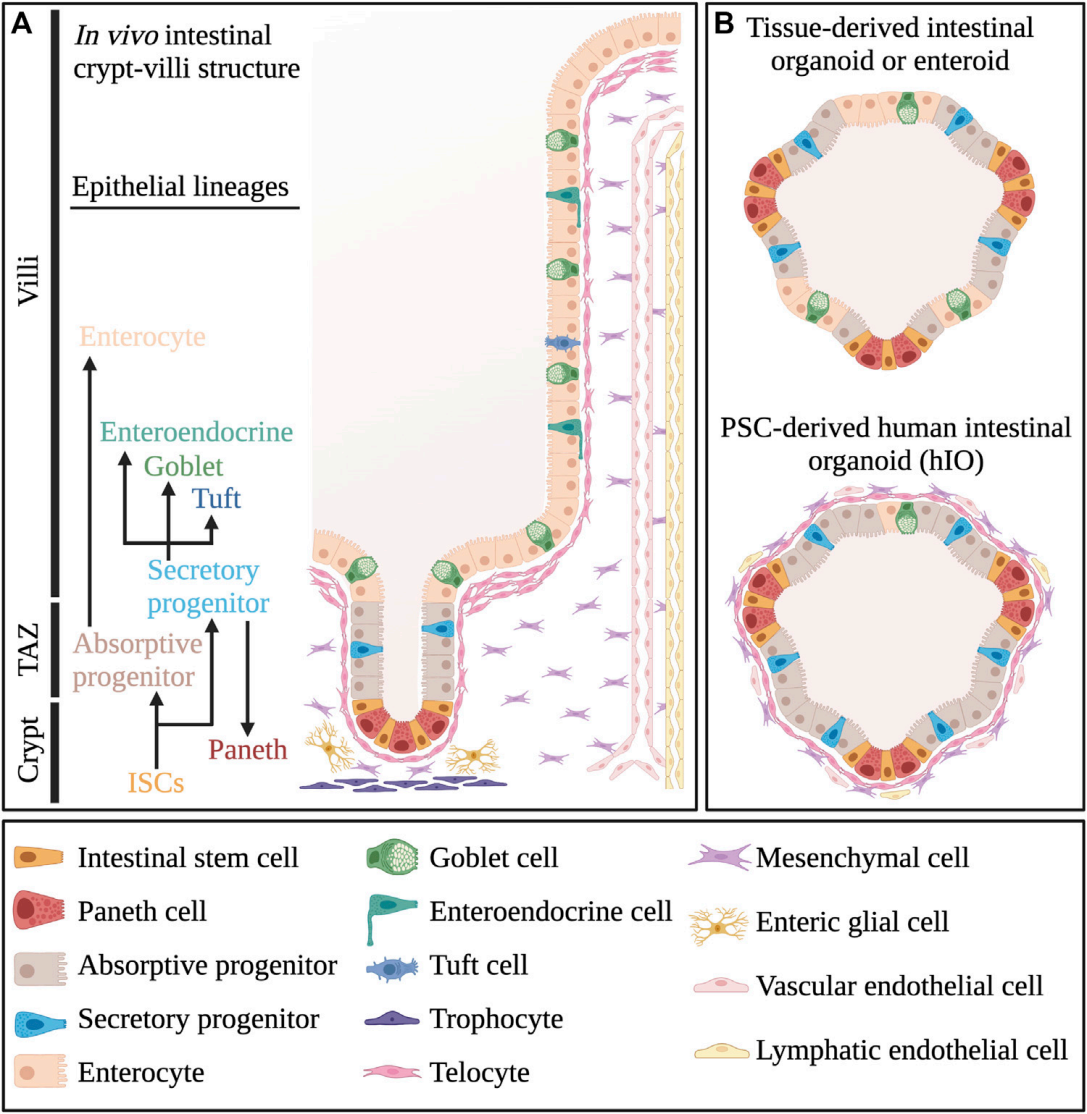
Cellular composition of intestinal tissue and organoids. **(A)** Different cell types that make up the crypt-villi structure *in vivo* small intestinal tissue. **(B)** Comparative overview of the cellular composition of pluripotent stem cell-derived human intestinal organoids or hIOs and tissue-derived organoids or enteroids. TAZ, Transit Amplifying Zone. Created with BioRender.com.

The small intestine is a major organ that regulates digestive function and nutrient absorption. It is structured into crypts and protruding finger-like domains called villi (Figure 1A). The whole intestine is lined with a highly specialized epithelium, supported by a complex underlying mesenchyme (Guiu and Jensen, 2015; Gehart and Clevers, 2019; McCarthy et al., 2020a). During homeostasis, the human intestinal epithelium undergoes complete renewal every 5–7 days (Barker, 2014). This highly regenerative nature is supported by multipotent intestinal stem cells (ISCs), residing at the bottom of the crypts (Barker et al., 2007). ISCs can be identified by the expression of the R-spondin (RSPO) receptor LGR5, which plays a major role in the regulation of WNT/RSPO-signalling (Clevers, 2013). The proliferative ISC population in the crypt is maintained by neutral competition for supportive signals, including high WNT concentration, Notch signals and EGF (Snippert et al., 2010; Sato et al., 2011a). These primarily originate from Paneth cells, differentiated secretory cells that migrate away from differentiated villi regions to become interspersed with ISCs (van Es et al., 2005; Sato et al., 2011a). Paneth cells are also specialised in the secretion of antimicrobial agents such as lysozyme, α-defensins and phospolipase-A2 (Porter et al., 2002). Nevertheless, ablation experiments in mice showed that Paneth cells are redundant (Kim et al., 2012; Van Es et al., 2019). While Notch-signalling can be provided by secretory lineage precursor cells, WNT and EGF signals are also supplied by multiple cell types of the subepithelial mesenchyme (Blumberg et al., 2008; Van Es et al., 2019; McCarthy et al., 2020a).

One specific CD81+/Pdgfra + mesenchymal subpopulation, termed trophocytes is primarily located below the crypt epithelium and can support mouse intestinal cells *in vitro* without any additional factors (McCarthy et al., 2020a) (Figure 1A). Aside from expression of canonical Wnt ligands and Wnt-promoting Rspo proteins, trophocytes are also typified by their expression of BMP inhibitors Gremlin 1 and Noggin (McCarthy et al., 2020a). Another subpopulation, Foxl1+/Pdgfra + subepithelial myofibroblasts (SEMFs), also termed telocytes, form a loosely interconnected sheat directly below the intestinal epithelium via their far-reaching cell extensions (Shoshkes-Carmel et al., 2018). Their distribution is non-uniform as they have higher concentrations near the villus base, as well as in villus tips (Shoshkes-Carmel et al., 2018). Depending on this distribution, telocytes express multiple Bmp factors and non-canonical Wnt factors which generally counteract canonical Wnt-signalling (Shoshkes-Carmel et al., 2018; McCarthy et al., 2020b). However, telocytes also express canonical Wnt agonists, and specific inhibition of Wnt secretion in mouse telocytes ultimately caused a dramatic reduction in ISC proliferation (Shoshkes-Carmel et al., 2018). The mesenchyme further harbours a large heterogenous population of Pdgfra^low^-mesenchymal cells which can express both Bmp factors, Wnt agonists and antagonists, further contributing to a complex mesenchymal environment (McCarthy et al., 2020a; Kim JE. et al., 2020). Specific subpopulations of Gfap + enteric glial cells (EGCs), localised near crypts, also provide key yet redundant Wnt factors to ISCs (Baghdadi et al., 2022). In case of injury however, these EGCs expand and are critical to efficient intestinal regeneration (Baghdadi et al., 2022). Various types of immune cells also reside in the intestinal mesenchyme and tissue-resident lymphoid cells have been implicated in the regulation of intestinal homeostasis and regeneration by secretion of interleukin factors (Lindemans et al., 2015; Zhu et al., 2019). Furthermore, endothelial cells may also be involved in ISC maintenance, as specific inhibition of endothelial cell apoptosis was sufficient to recover crypt homeostasis following irradiation damage (Paris et al., 2001). Lymphatic endothelial cells were also shown to express relatively high levels of Rspo3, contributing to the maintenance of intestinal homeostasis (Ogasawara et al., 2018).

Generally, factors secreted by Paneth cells and mesenchymal cells create a high WNT/RSPO-signalling environment towards the bottom of the crypt, while a high BMP-signalling environment is present towards the villi. Both gradients decrease towards the transition zone, creating a zonation with segregated environments that guide cell type identity (Sato and Clevers, 2013; Moor et al., 2018). As ISC daughter cells move from the bottom of the crypt to the villi domain, they move through these different molecular gradients, generating increasing maturation of specialized cell types (Moor et al., 2018; Shoshkes-Carmel et al., 2018; McCarthy et al., 2020a).

ISCs give rise to committed daughter cells, which differentiate into distinct absorptive and secretory lineages (Wright, 2000; Schuijers and Clevers, 2012). The absorptive lineage gives rise to nutrient-absorbing enterocytes, while the secretory lineage primarily generates diverse hormone-producing enteroendocrine cells, mucus-producing goblet cells and ISC-supporting Paneth cells (Clevers and Bevins, 2013; Beumer et al., 2018; Yang and Yu, 2021). Cells that reach the top of the villi go into apoptosis and are shed into the lumen. The relatively rapid turnover, combined with the changing molecular environments demands a high degree of plasticity from intestinal epithelial cells. This has recently been demonstrated in the mouse intestine, as all major intestinal cell types can de-differentiate to a stem cell state, in reaction to proper environmental cues. Following ablation of resident ISCs, cell-specific lineage tracing strategies demonstrated regeneration of ISCs and full crypt-villi structures from Alpi + enterocytes (Tetteh et al., 2016), Lyz + Paneth cells (Yu et al., 2018) and endocrine lineage precursor cells (Van Es et al., 2012; Barriga et al., 2017; Yan et al., 2017; Ayyaz et al., 2019). In the mouse fetal intestinal epithelium, all cells, irrespective of their location and differentiation status, actively contribute to the adult ISC pool (Guiu et al., 2019). Moreover, a recent study demonstrated that, following the loss of proliferative ISCs due to injury, de-differentiation processes are the main driving force in the generation of novel ISCs as opposed to a specialized injury-resistant reserve stem cell population (Murata et al., 2020). However, cells of the secretory lineage inherently have low proliferation rates, which may make them more resistant to routinely used forms of cell damage, such as irradiation or chemotherapy (Basak et al., 2014). This may make them more likely to be sources of de-differentiation. Interestingly, de-differentiation processes occur by a shift from an adult to a more fetal-like intestinal gene expression profile, particularly involving high YAP1-signalling (Gregorieff et al., 2015; Yui et al., 2018).

Due to limited tissue accessibility and ethical considerations, the elucidation of the cellular and molecular complexity of the intestine has relied on animal and *in vitro* model systems. In recent years, the development of three-dimensional organoids has transformed biomedical research fields, including tissue homeostasis, regeneration and stem cell differentiation (Leushacke and Barker, 2014). In this review, we discuss the advantages and disadvantages of different organoid models.

## Intestinal Organoids as an Improved Model to Study Intestinal Function

Mouse models have greatly enhanced our understanding of biological systems and have enabled the development of numerous therapeutic strategies. Most importantly, they have allowed us to study pathologies without involving human patients. However, insights from animal models cannot guarantee extrapolation to human tissues. For instance, the timeline of embryonic intestinal development in mice proceeds past birth, while human intestinal formation is largely completed during the second trimester (Guiu and Jensen, 2015). One major difference in these developmental schedules is the formation of Paneth cells, which in human appear during the first trimester while in mouse only by day 14 postnatally (Bry et al., 1994; Rumbo and Schiffrin, 2005). Small intestinal villi in mouse are taller than in human, even though the small intestine is proportionally about three times shorter in mice (Nguyen et al., 2015). Furthermore, EGF seems to have opposite effects on the maturation of *in vivo* intestinal epithelium in mouse and in human (Calvert et al., 1982; Ménard et al., 1988). Importantly, stable mouse organoid lines can be derived with minimal niche factors Rspo1, Noggin and EGF, while human organoids demand additional growth factors and inhibition of p38/MAPK pathways (Sato et al., 2011b; Fujii et al., 2018). In addition, studies on animal models remain encumbered by considerations of animal ethics, limiting their scalability to high-throughput use.

In the last decade, organoids have been increasingly used as *in vitro* models as they improve upon several of these shortcomings. Organoids are self-organizing three-dimensional structures, consisting of both multipotent tissue-specific stem cells, as well as their more differentiated progeny (Zachos et al., 2016). Similar to stem cell lines, they can be propagated virtually indefinitely, provided the right culture conditions are met (Sato et al., 2009; 2011b). While human intestinal organoids were the first human organoid types that were successfully established *in vitro*, many protocols have since been optimized for organoids of many other tissue types, including pancreas, liver, kidney, stomach and lung (Huch et al., 2013a, 2013b; Dye et al., 2015; Nishinakamura, 2019; Seidlitz et al., 2021). Organoids recapitulate *in vivo* tissue architecture, multiple cell type heterogeneity and interactions *in vitro*. Therefore, they will potentially model human tissue functionality and physiology more accurately than animal models or 2D models (Lancaster and Knoblich, 2014; Kim J. et al., 2020). For instance, when sufficiently matured, human intestinal organoids recapitulate budding crypt and villi domains, harbouring proliferative ISCs and progenitors, as well as differentiated enterocytes, goblet cells and Paneth cells, respectively (Onozato et al., 2021). Although organoids contain differentiated cell types, they can be cryopreserved and expanded relatively rapidly (Clinton and McWilliams-Koeppen, 2019). Organoids have proven to be highly versatile in experimental settings, as they are compatible with routinely used imaging techniques (Mahe et al., 2013; Dekkers et al., 2019), as well as live cell imaging (Zietek et al., 2015), assessment of metabolic activity (Berger et al., 2016), genome-wide gene expression and protein analyses (Lindeboom et al., 2018; Kip et al., 2021; Norkin et al., 2021) and gene editing techniques (Koo et al., 2012; Schwank et al., 2013; Schwank and Clevers, 2016; Driehuis and Clevers, 2017). Another major advantage of organoid models is their *in vitro* scalability to high-throughput assays, in combination with their recapitulation of 3D tissue functionality (Lukonin et al., 2020; Sugimoto and Sato, 2022). As such, organoids have been increasingly used to study intestinal function, to model disease phenotypes and for drug discovery screening (Fair et al., 2018; Frum and Spence, 2021).

## Pluripotent StemCell-Derived Human Intestinal Organoids vs. Tissue-Derived Enteroids

Different types of small intestinal organoids have been described and to distinguish them, a specific nomenclature has been proposed (Stelzner et al., 2012). Human small intestinal organoids can be generated by differentiation from human pluripotent stem cells (PSCs) or by derivation from isolated multipotent stem cells and progenitor cells present in *vivo* intestinal crypts. The former are conventionally referred to as human intestinal organoids (hIOs), while the latter are termed enteroids (Stelzner et al., 2012) (Figure 1B). Additionally, in the initial 24-h culture phase to generate enteroids, small circular structures are formed, which are referred to as enterospheres (Stelzner et al., 2012). The enterosphere phase is transversed by both enteroids and hIOs during their formation (Stelzner et al., 2012). In contrast to enteroids, hIOs recapitulate fetal small intestine developmental stages and are therefore an excellent tool for studying this process (Wells and Spence, 2014; Frum and Spence, 2021). However, they also differentiate into both epithelial and mesenchymal cells (Spence et al., 2011; Mahe et al., 2015). Multicellular organoids such as these are also sometimes referred to as “reconstituted intestinal organoids” (Stelzner et al., 2012). Thus, reconstituted intestinal organoids or hIOs may complicate our understanding of the molecular mechanisms occurring specifically between intestinal epithelial cells. Recently, Mithal and colleagues reported a novel strategy to generate mesenchyme-free organoids from human iPSCs (Mithal et al., 2020). On the other hand, the presence of mesenchymal cells does create an intestinal model which more closely resembles the *in vivo* tissue compared to organoids with only epithelial cells. If the goal is to study the co-dependency of both tissues during development, this is rather advantageous, as has been shown by the use of hIOs including mesenchymal cells to model fibrosis in ulcerative colitis (Sarvestani et al., 2021). However, upon establishment of hIO cultures, they largely retain fetal-like characteristics. This has been demonstrated by RNA-sequencing comparisons of hIOs with fetal and adult intestinal tissues and is likely caused by a lack of appropriate cues from the mesenchymal niche (Finkbeiner et al., 2015; McCarthy et al., 2020a).

In contrast, intestinal crypt-derived enteroids recapitulate adult intestinal architecture and genetic profiles of specific adult intestinal epithelial cell types more faithfully in mouse, with Lgr5+ stem cells residing in crypt-like zones and differentiated cells residing in inter-crypt zones (Schuijers and Clevers, 2012). Interestingly, transient depletion of proliferative cells reverts mouse intestinal organoids to a more fetal-like state (Gregorieff et al., 2015). Of note, fetal-like hIOs can be matured further by transplantation under the kidney capsule of immune-deficient mice (Watson et al., 2014). One major factor involved in this maturation may originate from endothelial cells, as post-transplanted mature organoids obtain extensive vascularization (Frum and Spence, 2021). Moreover, the co-culture of hIOs with endothelial cells also promotes organoid maturation (Palikuqi et al., 2020). Nevertheless, the directed differentiation process of PSCs towards intestinal organoids takes quite some time (2–3 months), after which hIOs still largely resemble fetal intestinal homeostasis (McCracken et al., 2011; Frum and Spence, 2021). Though recently, Onozato and colleagues described a method to enhance organoid maturation *in vitro*, generating crypt-like budding and expression of villin1 (Onozato et al., 2021). Conversely, enteroids do not have to undergo sequential differentiation steps and can therefore be generated much more rapidly. Furthermore, enteroids are capable of recapitulating intestinal epithelium beyond a fetal phenotype with a higher degree of maturation (Hofer and Lutolf, 2021). However, it is important to realize that full maturation is usually not achieved with enteroids either, due to conventional culture conditions that promote proliferation. Nevertheless, due to the lack of mesenchymal cells, enteroids provide a less complex model system compared to hIOs to study epithelial cell type-specific molecular functions.

## Limitations of Intestinal Organoids

Intestinal organoid technology has greatly enhanced research into tissue homeostasis, cell-to-cell interactions, differentiation, and physiology. However, several shortcomings still limit the model in its application to clinical research.

Firstly, while organoids are comprised of multiple cell types of the corresponding tissue, they still only include multipotent stem cells and their progeny. Enteroids therefore only include epithelial cell types. While hIOs do generate mesenchymal cells surrounding the epithelial cells, this is still rather limited compared to the *in vivo* intestine. Generally, both types of organoids lack complex mesenchymal heterogeneity and architecture, vasculature, neuronal connections and interaction with immune cells and the intestinal microbial flora. Several groups have therefore explored possible co-culture strategies to increase organoid complexity, including co-culture with endothelial (Palikuqi et al., 2020), mesenchymal (Stzepourginski et al., 2017), immune (Bar-Ephraim et al., 2020) and glial cells (Baghdadi et al., 2022), as well as microbial (Finkbeiner et al., 2012; Wilson et al., 2015; Fatehullah et al., 2016; Han et al., 2019; Barron et al., 2020) and viral interactions (Zhao et al., 2021). Yet, current co-culture systems are still relatively simple, only combining one or few cell types simultaneously. In the future, both hIO and enteroid models will benefit from efforts to increase complexity of co-culture systems.

Intestinal organoids inherently form a lumen-enclosed structure. This reduces access to the absorptive apical side and leads to accumulation of apoptotic cells inside organoids. This potentially confounds drug screening and microbial co-culture studies (Altay et al., 2019). Recently, several publications have described methods to generate reversion of the apical side to the outside of organoids (Co et al., 2019, 2021; Li et al., 2020; Stroulios et al., 2021). For instance, Co. and colleagues described how inhibition of the interaction between organoid epithelial cell β1-integrin receptors and the protein matrix in which they are cultured (such as Matrigel), caused spontaneous reversion of polarity (Co et al., 2019). Indeed, the localisation of the matrix used to create a supportive 3D environment, causes the apical-in polarity of organoids, similar to the extracellular matrix *in vivo*.

Conventional organoid culture mostly involves the embedding of organoids in Matrigel, a complex basement-membrane protein matrix, isolated from Engelbreth-Holm-Swarm mouse sarcoma tumor cells (Jee et al., 2019; Aisenbrey and Murphy, 2020). Matrigel provides essential ECM components to organoids, such as laminins, collagens and fibronectins (Jee et al., 2019; Aisenbrey and Murphy, 2020). Each of these components individually are capable of supporting the formation of ISC colonies, yet optimal differentiation to organoids was shown to require a delicate balancing of differential compositions (Gjorevski et al., 2016). However, due to Matrigel’s highly complex composition, inherent variability and mouse origin, recent efforts are focussing on the design and study of synthetic hydrogel alternatives (Rezakhani et al., 2021). In fact, mechanical properties such as stiffness and degradability are equally vital to the formation, maintenance and differentiation of organoids in culture (Gjorevski et al., 2016). As such, the mechanobiological properties of the protein matrix also influence the phenotype exhibited by organoids: 1) enteroids cultured in Matrigel generate budding structures that recapitulate *in vivo* behaviour (Sato et al., 2009); 2) organoids cultured in collagen I matrices acquire a foetal-like state mimicking the *in vivo* process of regeneration (Yui et al., 2018); 3) hanging drop cultures increase inter-organoid interactions, organoid fusion and can even induce continuous peristaltic movements (Ueda et al., 2010; Panek et al., 2018). Similarly, culture in floating collagen gels also generates fusion and self-organisation of intestinal organoids into tubular structures (Sachs et al., 2017). Nevertheless, as of yet, conventional organoid culture lacks the presence of *in vivo* growth factors, complex extracellular matrix composition, as well as fully optimized biomechanical properties of their environment (Guiu and Jensen, 2022). Importantly, intestinal organoid lines also possess a high degree of variability, as tissue heterogeneity (for enteroids), passaging methods, access to media components and positioning within 3D matrices impose a stochastic nature to their culture (Nikolaev et al., 2020).

Recently, novel approaches have been implemented aiming to meet several of these shortcomings. Experimentation on substrate matrix properties has allowed for improved monolayer culture of intestinal organoids, allowing access to the luminal side of intestinal cells and analysis of barrier integrity (Schweinlin et al., 2016; Altay et al., 2019). Chen and colleagues utilized a hollowed-out porous silk protein scaffold, onto which intestinal epithelial and myofibroblast cells could be seeded and maintained (Chen et al., 2015). This system recapitulated complex intestinal function and architecture, including mucus production and an accessible tubular lumen which allowed analysis of intraluminal oxygen tension and bacterial interaction (Chen et al., 2015). Future advancement of 3D bioprinting technology towards scaffold-free approaches will undoubtedly improve intestinal tissue modelling even further (Murphy and Atala, 2014; Wengerter et al., 2016). Organ-on-chip engineering has also recently been implemented to form perfusable *in vitro* mouse intestinal organoid structures, embedded in a pre-shaped matrix scaffold. Nikolaev and colleagues described the self-organization of intestinal cells into a perfusable tube-shaped structure with crypt and villi domains and differentiation into specialised intestinal cells, inside a preformed hydrogel scaffold on a chip (Nikolaev et al., 2020). Kasendra and colleagues created an intestinal and microvascular endothelial co-culture on a chip in parallel perfusable channels. Crypt and villi structures were formed with multilineage differentiation, while interfacing with the microvascular endothelial cells (Kasendra et al., 2018). Such perfusable models allow the sequential analysis of fluid samples to quantify nutrient digestion, mucus secretion and establishment of intestinal barrier function *in vitro* (Kasendra et al., 2018; Nikolaev et al., 2020). Overall, this technology has the potential to increase robustness of crypt-villi architecture formation, nutrient absorption, co-culture strategies, larger scale intestinal architecture, as well as morphogen gradients. Moreover, multi-organ co-culture chip strategies have already been explored to investigate higher order tissue interactions and barrier function (Maschmeyer et al., 2015a; 2015b). However, the increasing complexity of organ-on-chip approaches may also limit high-throughput scalability and escalate associated costs.

## Conclusion

**FIGURE 2:**
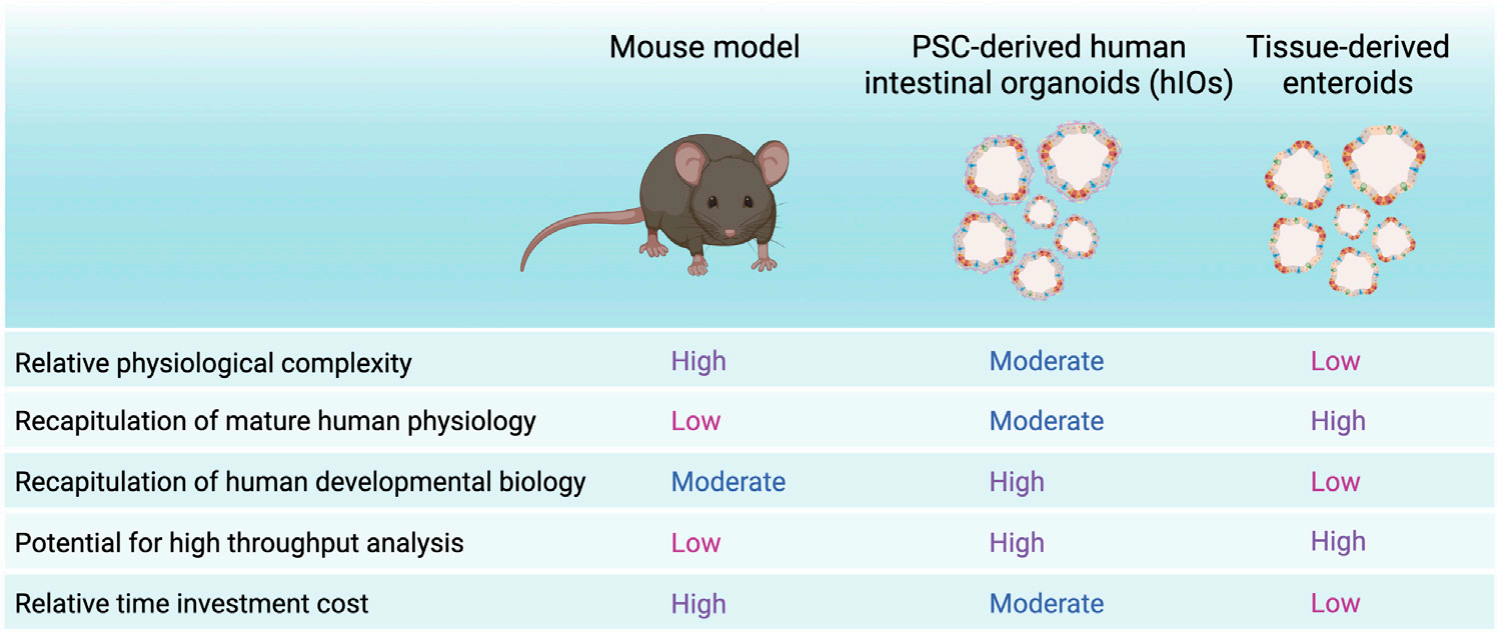
Strengths and weaknesses of hIOs, enteroids and mouse models. Summary of advantages and disadvantages of PSC-derived organoids or hIOs, tissue-derived organoids or enteroids and the mouse model. Created with BioRender.com.

Taken together, although human organoids provide attractive *in vitro* models, it is important to keep in mind they are still simplified culture models of a much more complex *in vivo* system (Figures 1A,B) (Guiu and Jensen, 2022). Yet, they provide a highly improved model compared to 2D culture approaches in terms of recapitulation of cell type-heterogeneity, cell type-interactions and 3D architecture. Compared to mouse models, intestinal organoids circumvent animal ethics considerations, allow high-throughput scalability and provide a human model intestinal functionality (Figure 2). As such, human intestinal organoids can represent a sufficiently complex *in vitro* model of intestinal tissue from fetal to adult human stages of development, which are otherwise difficult to access. Multiple recent reports have shown increased applicability and accuracy of organoid models, including maturation into budding organoids *in vitro*, outwards reversion of the apical side and induction of peristalsis. These improvements have increasingly benefitted potential future applications of hIOs, which could be used in a patient specific manner for drug screening and disease modelling purposes. Nevertheless, current organoid culture strategies still lack the complex interaction with *in vivo* growth factors, extracellular matrix composition and multi-organ physiology (Guiu and Jensen, 2022). Ultimately, future findings of interest from high-throughput screening studies using hIO or enteroid models should be validated *in vivo* in animal models, to provide information on the effects of the physiological environment, but also to assess whether findings from a simplified organoid structure can be recapitulated in the more complex *in vivo* intestinal tissue, albeit in an animal model.
